# Sperm quality in frozen beef and dairy bull semen

**DOI:** 10.1186/s13028-018-0396-2

**Published:** 2018-07-04

**Authors:** Jane Margaret Morrell, Andra Sabina Valeanu, Nils Lundeheim, Anders Johannisson

**Affiliations:** 1 Division of Reproduction, Department of Clinical Sciences, Faculty of Veterinary Medicine and Animal Science, Swedish University of Agricultural Sciences (SLU), 75007 Uppsala, Sweden; 2 University of Agricultural Sciences and Veterinary Medicine Iaşi, 3, Mihail Sadoveanu Alley, 700490 Iaşi, Romania; 3 Department of Animal Breeding and Genetics, Faculty of Veterinary Medicine and Animal Science, Swedish University of Agricultural Sciences, 75007 Uppsala, Sweden

**Keywords:** Chromatin integrity, Membrane integrity, Mitochondrial membrane potential, Motility, Reactive oxygen species

## Abstract

**Background:**

There is speculation that beef bull semen quality is inferior to that of dairy bulls although few scientific studies are available in the literature. The aim of this study was to evaluate sperm quality in beef bull semen and to determine which parameters could be indicative of fertility after insemination. Sperm quality, assessed by computer assisted sperm motility analysis and flow cytometric evaluation of membrane integrity, levels of reactive oxygen species, mitochondrial membrane potential, acrosome status and DNA fragmentation index, was evaluated in beef and dairy bull semen.

**Results:**

For beef bulls, normal morphology (r = 0.62, P < 0.05) and WOBBLE (r = 0.57, P < 0.05) were significantly correlated with 56-day non-return rate, whereas sperm quality was not significantly correlated with the fertility index score for dairy bulls. Membrane integrity (46 ± 8.0% versus 40 ± 11%, P < 0.05), normal morphology (87 ± 6% versus 76 ± 8%; P < 0.05), and high respiratory activity (52 ± 13 versus 12 ± 4%; P < 0.001) were higher for dairy bulls than for beef bulls. The DNA fragmentation index was lower for dairy bull spermatozoa than beef (3.8 ± 1.1% versus 6.1 ± 2.9%; P < 0.01), whereas some sperm kinematics were higher. Multivariate analysis indicated that type of bull (beef versus dairy) had an impact on sperm quality.

**Conclusions:**

Different assays of sperm quality may be needed for appropriate analysis of beef and dairy bull semen. These finding could be important for cattle breeding stations when evaluating semen quality.

## Background

Artificial insemination (AI) is one of the most effective tools available to dairy cattle producers to improve herd productivity and profitability [1]. Whereas more than 80% of dairy cows are artificially inseminated in e.g. North America or Europe, less than 5% of beef cows are bred by this method [1, 2]. The main reasons for this difference are that beef cows are managed under more extensive husbandry conditions than dairy cows, which makes oestrus detection problematic, and they may not be accustomed to being handled. Seasonal differences in fertility may occur and oestrus signs may not be as obvious as in dairy breeds; some of these issues may be overcome by hormonal manipulation of ovulation [3]. However, there is also speculation that beef bull semen quality is inferior to that of dairy bulls [4], although few scientific studies have been reported in the literature. Fertility is believed to vary up to 20% among bulls classified as having a satisfactory breeding soundness evaluation [5].

Although the market for beef bull semen for inseminating beef cows is generally stable, it is increasing in countries such as Brazil, where sales of beef semen have risen from approximately 3 million to nearly 12 million in less than 20 years [6]. Therefore, there is considerable potential to increase the use of AI in beef breeds in other countries. There is also a market for inseminating dairy heifers with beef bull semen to produce a crossbred calf with better beef-producing qualities than a purebred dairy calf, and an increasing interest in using sexed semen in beef cattle [7]. Thus, quality evaluation of beef bull semen is important. The aim of the present study was to make a retrospective analysis of sperm quality in commercial doses of frozen beef bull semen and to identify some parameters of sperm quality that could be used as indicators of potential fertility. A further aim was to identify where potential differences in sperm quality occur between beef and dairy bull semen.

## Methods

### Semen

Commercial straws of frozen semen from 14 beef bulls (6 Limousin, 3 Charolais, 2 Simmental, 2 Hereford, 1 Angus) frozen in Triladyl extender (Minitüb, Tiefenbach, Germany) were available from the Estonian Animal Breeders Association, Rapla, Estonia. Semen from 19 dairy bulls (10 Swedish Red and 9 Holstein) and 4 beef bulls (2 Limousin, 1 Charolais and 1 Blonde D’Aquitaine) frozen in Andromed (Minitüb) was supplied by VikingGenetics, Skara, Sweden. The straws were thawed in a water bath at 37 °C for 12 s.

### Sperm concentration

A Nucleocounter SP-100 (Chemometec) was used to measure sperm concentration. Aliquots (50 µL) of frozen-thawed semen were diluted with 5 mL S100 reagent to permeabilise the sperm membranes and a cassette containing propidium iodide (PI) was loaded with the mixture. The cassette was inserted into the fluorescence reader, which displayed the sperm concentration after approximately 30 s.

### Sperm morphology assessment

Wet smears were air dried and stained with carbolfuchsin-eosin, [8] for evaluation of five hundred spermatozoa at 1000× magnification under oil immersion. Additionally, sperm head morphology of 200 spermatozoa was assessed at 1000× magnification using aliquots of semen fixed in formol-saline. The sperm morphology assessment was carried out by skilled personnel at the Swedish Sperm Reference Laboratory, Swedish University of Agricultural Sciences (SLU). The proportions of morphological abnormalities such as proximal cytoplasmic droplets, acrosome defects, detached heads, nuclear pouches and tail defects were calculated. Normal morphology was calculated as 100 − % spermatozoa with abnormal morphology.

### Computer assisted semen analysis (CASA)

Sperm kinematics were assessed objectively using a CASA system, consisting of a phase-contrast Olympus BX 51 microscope (Olympus, Japan) connected to the SpermVision™ (Minitüb, Tiefenbach, Germany). An aliquot (5 µL) of semen was deposited on a warmed microscope slide at 38 °C and covered with a coverslip (18 × 18 mm). Sperm images in 8 fields were digitized for analysis of the kinematic patterns using the SpermVision™ software. The mean values were calculated for each of the following parameters based on approximately 1000 spermatozoa [9]: total motility (TM%), progressive motility (PM %), VCL (velocity curved line, µm/s), VSL (velocity straight line, µm/s), VAP (velocity average path, µm/s), ALH (amplitude of lateral head displacement, µm), BCF (beat cross frequency, Hz) and the ratios STR (straightness, VSL/VAP), LIN (linearity, VSL/VCL), and WOB (wobble, VAP/VCL).

### Plasma membrane integrity

Membrane integrity was analysed by flow cytometry after SYBR14-PI staining [10]. Aliquots of thawed semen were extended to a concentration of approximately 2 × 10^6^ spermatozoa/mL for staining with the Live-Dead Sperm Viability KIT L-7011 (Invitrogen). An aliquot (300 µL) of the sperm suspension was stained with 0.6 µL SYBR14 (final stain concentration 0.02 µM) and 3 µL PI (final stain concentration 12 µM), and incubated for 10 min at 38 °C. The stained samples were evaluated using a BD LSR flow cytometer (Beckon Dickinson, San José, CA, USA), the excitation being induced by an argon-ion laser (488 nm). Green fluorescence was detected with a FL 1 band-pass filter (530/30 nm) while red fluorescence was measured using a FL 3 long-pass filter (> 670 nm). A total of 50,000 sperm-specific events was evaluated and classified as membrane intact (SYBR14 positive; live), membrane damaged (dying: SYBR14 positive, PI positive; dead: SYBR14 negative, PI positive).

### Reactive oxygen species (ROS)

Reactive oxygen species were determined using a modification of the protocol described by Guthrie and Welch [11], in which the spermatozoa were stained with hydroethidine (HE; Molecular Probes, Inc.), 2′,7′-dichlorodihydrofluorescein diacetate (DCFDA; Molecular Probes, Inc.) and Hoechst 33258 (HO). The ROS superoxide (SO·) and hydrogen peroxide (H_2_O_2_) were determined using HE and DCFDA, respectively, while HO was added to enable live and dead cells to be differentiated.

After adjusting the sperm concentration to approximately 2 × 10^6^ spermatozoa/mL, the spermatozoa were stained as follows: two aliquots (300 µL) of each sample were stained with 9 µL of HO (40 µM), 9 µL HE (40 µM) and 9 µL DCFDA, (2 mM). In addition, 3 µL Menadione (MEN, 20 mM) were added to one of the aliquots to stimulate ROS production. After mixing gently, the samples were incubated at 38 °C for 30 min.

The analysis was carried out using the BD LSR flow cytometer (Beckon Dickinson, San José, CA, USA). Excitation was achieved with an argon-ion laser (488 nm) and a HeCd laser (325 nm); green fluorescence was detected with a FL1 band pass filter (530/30 nm), red fluorescence with a FL3 long pass filter (> 670 nm) and blue fluorescence with a FL4 band pass filter (510/20 nm). In total, 30,000 sperm-specific events were evaluated and classified as the proportions of: Live, Superoxide negative; Live, Superoxide positive; Dead superoxide positive; Live, H_2_O_2_ negative; Live, H_2_O_2_ positive; Dead, H_2_O_2_ negative; Dead, H_2_O_2_ positive (%).

### Mitochondrial membrane status

Aliquots of semen (300 µL) at a sperm concentration of approximately 2.5 × 10^6^/mL, were mixed with 1.2 µL 5,5′,6,6′-tetrachloro-1,1′,3,3′-tetraethylbenzimidazolylcarbocyanineiodide (JC-1) (stock 3 mM) and incubated for 40 min at 38 °C as described by Garner and Thomas [12]. The JC-1 fluorescence was measured in the FL1 (530/30 nm) and FL2 (585 nm) channels of the flow cytometer. In total, 10,000 cells were evaluated and classified in two categories: high respiratory activity (orange fluorescence) and low respiratory activity (green fluorescence).

### Sperm chromatin structure assay

Chromatin integrity was evaluated using the metachromatic dye acridine orange (AO). The DNA fragmentation index (%DFI) was expressed as the proportion of cells with denatured, single stranded DNA (red fluorescence) out of the total population (stable, double stranded DNA [green fluorescence] + single stranded DNA). An aliquot (20 µL) of thawed semen was mixed 1:1 (v/v) with TNE buffer, snap-frozen in liquid nitrogen and stored at − 80 °C. For analysis, samples were thawed on ice, and an aliquot (10 µL) was mixed with 90 µL of TNE followed by 200 µL of acid-detergent solution. After 30 s, AO (600 µL) was added and the sample was analyzed within 3–5 min using a FACStar Plus Flow cytometer with settings and software as described previously by Morrell et al. [13].

### Acrosome status

Sperm acrosome status and cell viability were assessed using fluorescein isothiocyanate-PNA (FITC-PNA) labeling and PI, respectively. An aliquot (300 µL) of semen, previously diluted to a sperm concentration of 2 × 10^6^/mL, was mixed with 3 µL of FITC-PNA (previously diluted tenfold with buffer enriched with 10 mM Calcium and Magnesium) and 3 µL of PI, and was incubated at 38 °C for 10 min [14, 15]. The fluorescence from 50,000 sperm-events was recorded after gating out the non-sperm events. PI was detected using the FL 3 long-pass filter (> 670 nm), while FITC-PNA fluorescence was detected at 515–545 nm in the FL1. The sperm subpopulations were categorized as live acrosome reacted, dead acrosome damaged, live non-reacted acrosome and dead non-reacted acrosome (%).

### Fertility

The 56-day non-return rate after first insemination in 130 to > 1000 cows was available for the beef bulls. For the 19 dairy bulls, a fertility index score was provided for inseminations in > 1000 cows, which had been calculated by adjusting the non-return rate to account for factors such as the age and parity of the female, farm location, inseminator etc.

### Statistical analysis

The statistical analyses were performed using the SAS software (ver. 9.3; SAS Inst.). To evaluate the difference between the two groups of bulls, analysis of variance (PROC GLM) was applied, using a statistical model including the effect of breed. The relationships between sperm quality and fertility (either the 56-day non-return rate or the fertility index score) were analysed within bull type (beef or dairy) using Spearman rank correlation. Only bulls with more than 130 cows were included in the correlation analysis. In all cases, P < 0.05 was considered to be significant.

To investigate the interaction of extender and type of semen i.e. beef or dairy bull semen, multivariate analysis was performed with Partial Least Squares Regression (PLS) using Simca software (version 14; MKS Data Analytics Solutions, Umeå, Sweden), as described on the website http://onlinelibrary.wiley.com/doi/10.1002/cem.1006/full.

## Results

### Sperm concentration

The mean concentration (± SD) was 88 ± 20 × 10^6^/mL and 55 ± 19 × 10^6^/mL for beef and dairy bull semen, respectively (P < 0.001).

### Sperm morphology

Normal morphology for beef and dairy bulls was 76 ± 8% and 87 ± 6%, respectively (P < 0.05).

### CASA kinematics

Except for total motility, progressive motility and BCF, the sperm kinematics were significantly different for the two types of semen (Table 1). Most kinematics were higher for dairy bull spermatozoa than for beef bull spermatozoa, with the exception of STR, LIN and WOB which were higher for beef than for dairy.

**Table 1:** Sperm motility parameters (mean ± SD) measured by computer assisted sperm analysis in semen from beef to dairy bulls (n = 37)

Parameters	Beef (n = 17)	Dairy (n = 20)	Significance
Motility (%)	64 ± 14	59 ± 14	NS
Progressive motility (%)	58 ± 13	55.6 ± 14	NS
Hyperactivity (%)	6 ± 3	11 ± 5	P < 0.01
VAP (μm/s)	52 ± 4	65 ± 8	P < 0.001
VCL (μm/s)	87 ± 8	128 ± 18	P < 0.001
VSL (μm/s)	39 ± 3	45 ± 7	P < 0.01
STR	0.75 ± 0.03	0.70 ± 0.05	P < 0.001
LIN	0.44 ± 0.02	0.35 ± 0.04	P < 0.001
WOB	0.59 ± 0.01	0.50 ± 0.03	P < 0.001
ALH (μm)	4.1 ± 0.6	4.98 ± 0.5	P < 0.001
BCF (Hz)	23 ± 1.4	22 ± 2.5	NS

*NS* not significant (P > 0.05)

### Membrane integrity

Beef bull semen contained a lower proportion of membrane intact spermatozoa (Table 2) than dairy bull semen (40 ± 11% versus 46 ± 8%, respectively; P = 0.053).

**Table 2:** Living, dying, dead sperm cells, %DFI, acrosome status, and mitochondrial membrane potential in frozen-thawed AI doses (mean ± SD) from beef to dairy bulls (n = 37)

Parameter	Beef (n = 17)	Dairy (n = 20)	Significance
Living (%)	40 ± 11	46 ± 8	Trend P = 0.053
Dying (%)	6 ± 3	5 ± 3	NS
Dead (%)	55 ± 14	49 ± 8	NS
% DFI	6 ± 3	4 ± 1	P < 0.01
Live not acrosome reacted (%)	59 ± 12	52 ± 8	P < 0.05
Dead not acrosome reacted (%)	18 ± 12	32 ± 8	P < 0.001
Live acrosome reacted (%)	0.3 ± 0.3	0.3 ± 0.2	NS
Dead damaged (%)	21 ± 11	17 ± 10	P < 0.001
High respiratory activity (%)	36 ± 10	58 ± 12	P < 0.001
Low respiratory activity (%)	64 ± 10	42 ± 12	P < 0.001

*NS* not significant (P > 0.05)

### Oxidative stress

In both control samples and samples stimulated with menadione (Table 3), there was a significant difference (P < 0.001) between dairy and beef bulls in live superoxide negative and live superoxide positive spermatozoa (P < 0.001). Additionally, in the samples not stimulated with menadione, there was a trend towards significance (P < 0.08) for dead H_2_O_2_ negative sperm cells between the two types of bulls, although no significant differences were observed for the other categories of ROS. In samples stimulated with menadione, trends towards significance were seen for live H_2_O_2_ positive spermatozoa (P < 0.055) and dead, H_2_O_2_ negative spermatozoa (P < 0.066) between the two types of bulls.

**Table 3:** ROS-production (mean ± SD) in beef and dairy bull semen (n = 17 and 20 respectively)

ROS category	Beef	Dairy	Beef + MEN	Dairy + MEN
Live superoxide negative (%)	57 ± 18^a^	35 ± 14^a^	62 ± 23^b^	37 ± 12^b^
Live superoxide positive (%)	8 ± 3^a^	25 ± 7^a^	6 ± 6^b^	24 ± 10^b^
Dead superoxide positive (%)	36 ± 17	40 ± 11	32 ± 20	39 ± 9
Live hydrogen peroxide negative (%)	61 ± 23	61 ± 11	68 ± 20	62 ± 9
Live hydrogen peroxide positive (%)	4 ± 14	0.2 ± 0.3	0.6 ± 1.2*	0.1 ± 0.1*
Dead hydrogen peroxide negative (%)	31 ± 16*	39 ± 11*	30 ± 19*	39 ± 9*
Dead hydrogen peroxide positive (%)	4 ± 11	0.08 ± 0.2	1.2 ± 2.9	0.05 ± 0.08

Same superscripts within a row indicate significant difference (P < 0.001)

*MEN* menadione

* Denotes trend towards significance: for live hydrogen peroxide positive with menadione P < 0.055; for dead hydrogen peroxide negative P < 0.08; for dead hydrogen peroxide negative with menadione P < 0.066

### Acrosome integrity

**Fig. 1:**
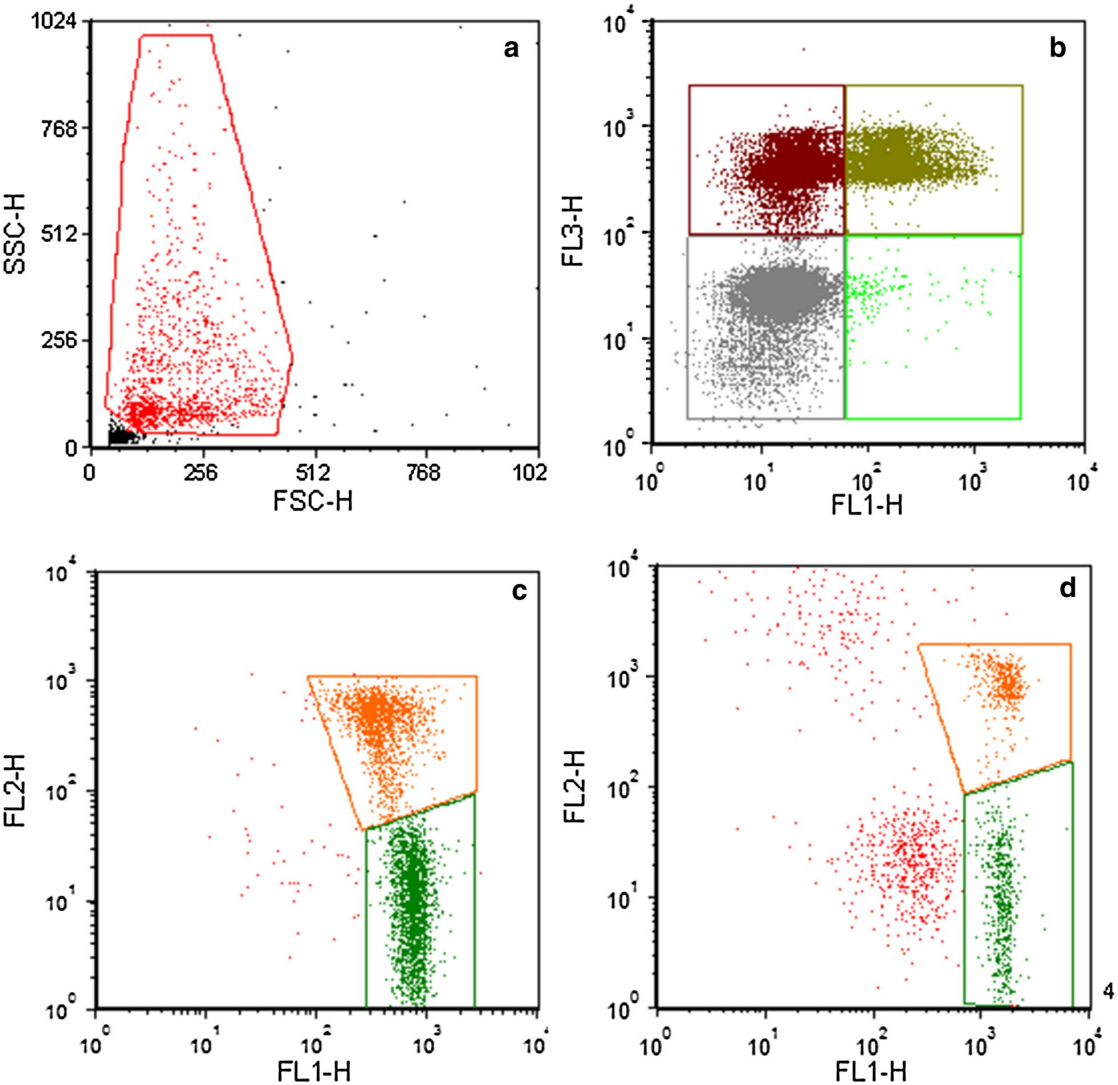
Flow cytometry data: **a** FSC-SSC pattern obtained as well as the gate used to select spermatozoa for further analysis steps; **b** shows the regions of analysis used in the acrosome status assay. Lower right: live, reacted, green; Upper right: dead damaged, olive; Lower left: live, non-reacted, grey; Upper left: dead, non-reacted, dark red; **c**, **d** results from analysis of mitochondrial membrane status of spermatozoa from a dairy bull and a beef bull, respectively. Indicated are the regions of analysis for spermatozoa with high MMP (orange) and low MMP (green). Events inside the gate for spermatozoa, but outside the regions of analysis, are shown in red

The FSC-SSC pattern obtained and the regions of analysis used in the assay for acrosome status are shown in Fig. 1a, b, respectively. There were significant differences (Table 2) in the proportions of live acrosome intact (59 ± 12 versus 52 ± 8; P < 0.05), dead acrosome intact (18 ± 12 versus 32 ± 8; P < 0.001), and dead acrosome damaged sperm cells between beef and dairy semen (21 ± 11 versus 17 ± 10%; P < 0.001).

### Mitochondrial potential

Figure 1c, d show the FSC-SSC pattern obtained with JC-1 staining of dairy and beef bulls, respectively, as well as the gate used to select spermatozoa for further analysis steps. For beef bulls, the proportion of sperm with high respiratory activity (Table 2) was 36 ± 10%, significantly lower (P < 0.001) than for dairy bulls (52 ± 12%). The proportion of sperm with low respiratory activity was higher for beef bulls than dairy bulls (64 ± 10% versus 42 ± 12%; P < 0.001).

### Sperm chromatin structure assay

The %DFI was significantly higher (P < 0.01) for beef bulls (6.1 ± 2.9) than for dairy bulls (3.8 ± 1.1).

### Correlations of sperm quality with fertility

**Fig. 2:**
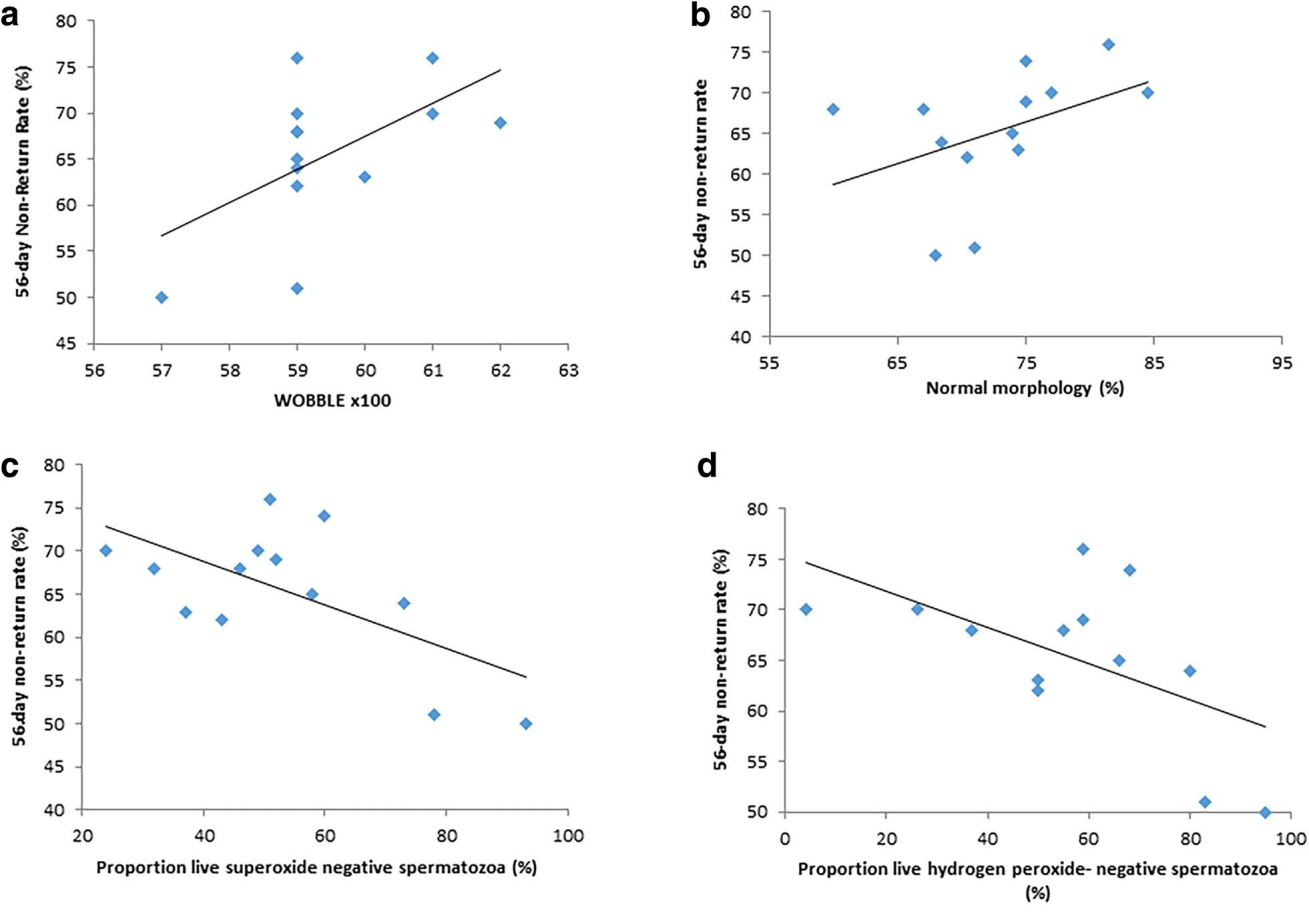
Relationship between various sperm characteristics and 56-day non-return rate for beef bulls (n = 13). **a** WOBBLE; **b** normal morphology; **c** live superoxide negative; **d** live hydrogen peroxide negative

For beef bulls, the kinematic WOBBLE (Fig. 2a) was significantly correlated with 56-day non-return rate (r = 0.59, P < 0.05), as was normal morphology (Fig. 2b; r = 0.517, P = 0.059). In addition, there were negative relationships between live superoxide-negative spermatozoa and 56-day non-return rate (r = − 0.63, P < 0.05, Fig. 2c), and between live hydrogen peroxide-negative spermatozoa and 56-day non-return rate (r = − 0.62, P < 0.05; Fig. 2d). In contrast, for dairy bulls there were no significant relationships between fertility index and sperm quality.

### Multivariate analysis

**Fig. 3:**
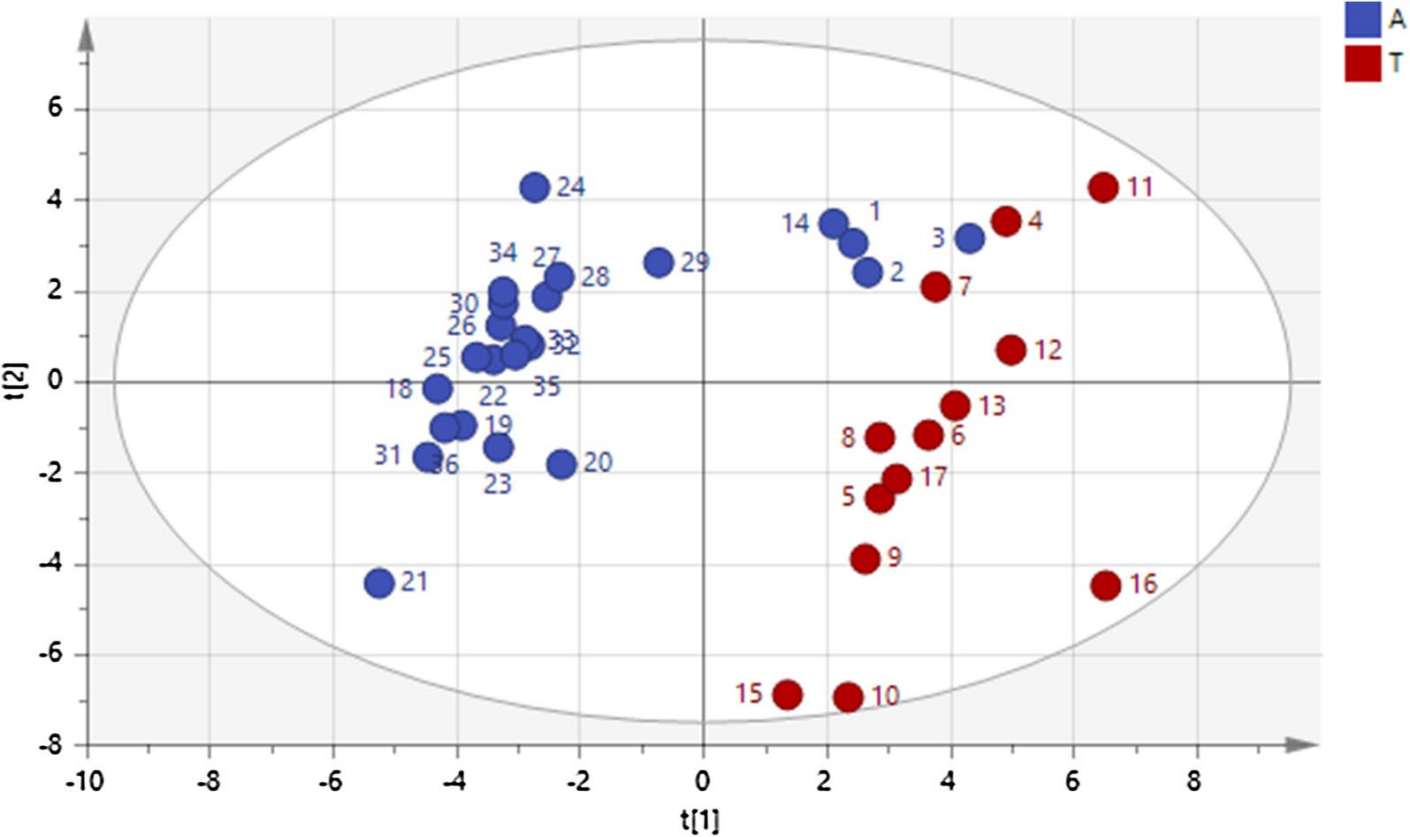
Scatter plot of beef and dairy bulls according to sperm quality (n = 37)

**Fig. 4:**
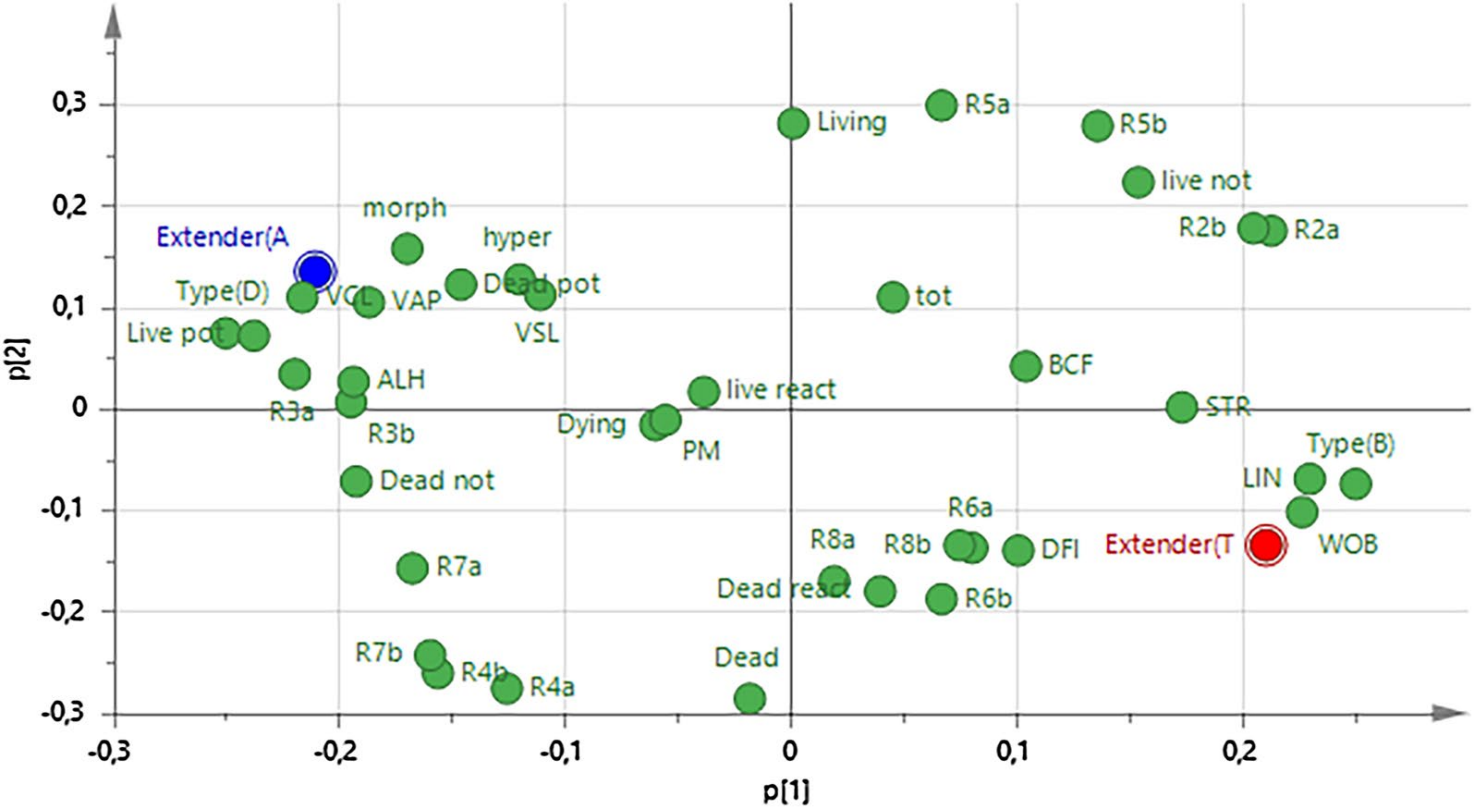
Partial Least Squares loading plot showing the relationship of breed to the various parameters of sperm quality. Type B and D refer to beef and dairy bulls respectively, Extender A and T refer to Andromed and Triladyl, respectively; *morph* morphology, *living, dead and dying* refer to membrane integrity, *hyper* hypermotility, *VCL* curvilinear velocity, *VSL* straight line velocity, *VAP* velocity of the average path, *STR* straightness, *LIN* linearity, *WOB* wobble, *ALH* amplitude of lateral head deviation, *BCF* beat cross frequency; high and low potential: high and low mitochondrial membrane potential; *live react, dead react, live not and dead not* acrosome status (acrosome reacted or not reacted) in relation to viability, *R2* live superoxide negative, *R3* live superoxide positive, *R4* dead superoxide positive, *R5* live hydrogen peroxide negative, *R6* live hydrogen peroxide positive, *R7* dead hydrogen peroxide negative, *R8* dead hydrogen peroxide positive; a or b in connection with R2-R8 refer to non-stimulated and stimulated with menadione, respectively

The PLS scatter plot showing the distribution of the individual bulls according to type (beef or dairy) and extender is shown in Fig. 3. The values for beef bulls clustered on the right side of the scatter plot, irrespective of extender, whereas those for dairy bulls clustered on the left. The PLS loading plot of the variables obtained by analysing type of bull (beef versus dairy) against all other variables (Fig. 4) showed that normal morphology, mitochondrial membrane potential, live reacted acrosomes, and the kinematics VCL, VSL, VAP and ALH clustered together with dairy bull semen and Andromed extender. In contrast, %DFI, categories of hydrogen peroxide producing spermatozoa, Linearity and Wobble, and dead reacted acrosomes clustered together with beef bull semen and Triladyl. The clusters appeared in opposite quadrants on the plot (top left for variables associated with dairy bulls and bottom right for those associated with beef bulls), indicating that they are different.

## Discussion

In this study, some interesting relationships between sperm quality and pregnancy rates were observed, not the least the fact that sperm motility (the most widely-used assessment of sperm quality) was not significantly related to pregnancy rate for these bulls. Significant differences in sperm quality between the two types of bulls were seen, as were correlations between sperm quality and pregnancy rate for beef bulls (namely WOBBLE and normal morphology), whereas there were no links between sperm quality parameters and fertility for the dairy bulls. There were significant differences between sperm motility patterns in semen from beef and dairy bulls; however, the semen was frozen in different extenders on the two commercial cattle stations, which could have affected the assessment of kinematics. Multivariate analysis showed that the sperm quality of beef bull semen frozen in Andromed was more similar to that of beef bull semen frozen in Triladyl than to sperm quality of dairy bull semen frozen in Andromed. Therefore, we deduce that type of bull semen (beef versus dairy) has more effect on sperm quality than the extender itself (or differences in the semen handling and freezing protocols on the two commercial semen stations), and therefore it is justifiable to combine the data for all of the beef bulls in this study, regardless of extender.

The sperm concentration in the straws was different for beef and dairy semen. However, this should not have affected the motility analysis since the sperm concentration was not too high to be analysed by the CASA instrument. Sperm concentration was adjusted to the same levels in all samples for staining in the flow cytometric analyses. However, sperm concentration could be important depending on the type of abnormality that is present. So-called compensable abnormalities are ones that impede the ability of the spermatozoa to reach the oviducts or to interact with the oocyte [16]; increasing the sperm concentration improves the chances of conception by increasing the number of normal spermatozoa that are present until the required threshold for conception is reached. Although originally described for some morphological defects [17], the idea of other sperm characteristics being compensable has gained ground e.g. membrane integrity, mitochondrial membrane potential, whereas sperm chromatin damage, protamine status etc. are considered to be non-compensable [16], because increasing the number of spermatozoa inseminated does not increase the chances of pregnancy.

According to a study in fresh bull semen [18], high sperm concentration may cause an increase in oxidative stress, with negative effects on cell viability. Superoxide production was significantly less in the semen from beef bulls than from dairy bulls but the extenders used may contain differing amounts of antioxidants, which would remove these ROS. It was proposed that ROS damage sperm DNA, at least in human patients with oligoasthenozoospermia [19], but this may not be the case in semen from normal bulls, especially where extenders containing antioxidants are used. In the beef bulls in the present study, there was a negative relationship between live spermatozoa with no superoxide or hydrogen peroxide production and fertility. These results indicate that lack of ROS-production could indicate low metabolic activity in these thawed spermatozoa, and that sperm cells with low metabolism did not result in pregnancies.

Reactive oxygen species are believed to cause decreased motility, decreased viability, and a number of morphological defects, particularly in the mid-piece. Although viability was superior in the dairy bulls in the present study, no significant differences were observed in total and progressive motility between breed types. These results are in contrast to those obtained by Fiaz et al. [20], who observed significantly higher total mobility (P < 0.05) in dairy bulls compared with beef ones. There were significant differences in all velocity kinematics in our study, being higher in spermatozoa from dairy bulls although they showed lower linearity (P < 0.001) than spermatozoa from beef bulls. In contrast, beef bull spermatozoa showed a higher beat cross-frequency and lower amplitude of lateral head displacement than dairy bull spermatozoa. Our results are in contrast to those of Hoflack et al. [21], who reported that Holstein bull spermatozoa showed superior total motility, progressive motility and linearity than spermatozoa from Belgian Blue bulls. However, as previously stated, the extenders used for dairy and beef bull semen were different (Andromed and Triladyl, respectively), which may have had an impact on sperm motility assessment. Zhang et al. [22] suggested that an improvement of boar sperm motility in extender containing soybean milk could be due to the lower viscosity and less debris compared to egg yolk.

A comparative study on AndroMed^®^, Bioxcell^®^ and Triladyl^®^ extender for cryopreservation of bull semen [23] showed that total and progressive motility were better in the ejaculates processed with Andromed, compared to those processed with Triladyl or Bioxcell. For sperm viability, however, significantly better results were obtained using Triladyl compared to Andromed and Bioxcell. In contrast, mitochondrial membrane potential and the DNA fragmentation index are improved in the soy-based extender compared to the milk-based extender during liquid storage [24]. According to the study that we conducted, both DNA fragmentation and low mitochondrial membrane potential were significantly higher for beef bull spermatozoa (extended with Triladyl) than for dairy bull spermatozoa (in Andromed).

The effect of bull type on other sperm quality parameters such as sperm morphology, may be explained by breed differences in adaptability to environmental conditions [25] and scrotal circumference. Studies showed a significant positive correlation (P < 0.05) between primary morphological defects and sperm DNA fragmentation in Holstein-Frisian bulls [26], although such a relationship was not seen in our study (data not shown). However, in the present study, dairy bull spermatozoa showed fewer primary morphological defects than beef bulls, and a statistically significant difference (P < 0.01) was observed between breeds for the proportion of sperm cells with fragmented DNA. These results correspond with other studies where lower sperm DNA fragmentation was found in dairy breeds than beef breeds [27]. Damage to sperm DNA does not impede oocyte fertilization or completion of early stages of cleavage but may block blastocyst formation by inducing an apoptosis-like phenomenon [28]. Sperm DNA lesions have been correlated with deficiencies in embryonic development [29, 30]. The significant differences between dairy and beef bulls for plasma membrane integrity, acrosomal integrity and mitochondrial function could indicate potentially higher fertility for the dairy semen since these parameters were found to be related to pregnancy rate in Nellore cows [31], although they were not correlated with higher fertility index score in our study.

Interestingly, multivariate analysis indicated that the type of bull (beef or dairy) had more of an effect on sperm quality than the extender. Since different extenders were used on the two commercial semen stations, the variable “extender” could also reflect differences in sperm handling procedures or freezing protocols. However, regardless of such potential sources of variation, beef bulls clustered together rather than clustering according to extender.

The observed differences in sperm quality between beef and dairy breeds may be due in part to intensive selection among dairy sires both in terms of production traits in their offspring and in sperm quality. The relationships between sperm quality and pregnancy rate for the beef bulls are interesting and warrant further study with a larger number of bulls. It would also be interesting to study possible breed differences among beef breeds, especially since a breed effect has been observed in dairy bulls [32]. The lack of significant correlations between sperm quality parameters and the dairy bull fertility index may be due to the use of an adjusted score instead of the unadjusted 56-day non-return rate, or to the relatively similar values in fertility index score for these bulls. It would be interesting to include bulls with higher and lower fertility scores than the ones used here. Unfortunately, it was only possible to use retrospective non-return data to look for associations with various parameters of sperm quality; thus, no account is taken of differences in female factors or herd management [33], which are likely to be different between dairy and beef cows. Nevertheless, the findings presented here are of interest in the context of the questions posed, namely whether some parameters of sperm quality could be identified that might be useful as indicators of fertility for beef bulls, and whether different parameters are needed for beef bull semen than for dairy bull semen.

## Conclusions

Significant relationships were seen between normal morphology and the 56-day non-return rate for beef bulls, and also for WOBBLE and the 56-day non-return rate, although no correlations were found between sperm quality and the fertility index score for dairy bulls. There were some differences in sperm quality between dairy and beef bulls. Multivariate analysis indicated that type of bull (beef versus dairy) had more effect on sperm quality than the extender used. Thus, different parameters of sperm quality may be needed as indicators of fertility for the two types of bull. These finding could be important for cattle breeding stations when evaluating semen quality.
